# Efficient Improvement in Fracture Toughness of Laminated Composite by Interleaving Functionalized Nanofibers

**DOI:** 10.3390/polym13152509

**Published:** 2021-07-29

**Authors:** Seyed Mohammad Javad Razavi, Rasoul Esmaeely Neisiany, Moe Razavi, Afsaneh Fakhar, Vigneshwaran Shanmugam, Vasudevan Alagumalai, Michael Försth, Gabriel Sas, Oisik Das

**Affiliations:** 1 Department of Mechanical and Industrial Engineering, Norwegian University of Science and Technology (NTNU), Richard Birkelands vei 2b, 7491 Trondheim, Norway; 2 Department of Materials and Polymer Engineering, Faculty of Engineering, Hakim Sabzevari University, Sabzevar 9617976487, Iran; 3 Department of Chemical Engineering, Isfahan University of Technology, Isfahan 8415683111, Iran; mo.razavi@yahoo.com (M.R.); a.fakhar@iut.ac.ir (A.F.); 4 Department of Mechanical Engineering, Saveetha Institute of Medical and Technical Sciences, Saveetha School of Engineering, Chennai 602105, India; vasudevana.sse@saveetha.com; 5 Department of Civil, Structural and Fire Engineering Division, Environmental and Natural Resources Engineering, Luleå University of Technology, 97187 Luleå, Sweden; michael.forsth@ltu.se (M.F.); gabriel.sas@ltu.se (G.S.)

**Keywords:** carbon/epoxy structural composites, fracture toughness, nanofibers, functionalization

## Abstract

Functionalized polyacrylonitrile (PAN) nanofibers were used in the present investigation to enhance the fracture behavior of carbon epoxy composite in order to prevent delamination if any crack propagates in the resin rich area. The main intent of this investigation was to analyze the efficiency of PAN nanofiber as a reinforcing agent for the carbon fiber-based epoxy structural composite. The composites were fabricated with stacked unidirectional carbon fibers and the PAN powder was functionalized with glycidyl methacrylate (GMA) and then used as reinforcement. The fabricated composites’ fracture behavior was analyzed through a double cantilever beam test and the energy release rate of the composites was investigated. The neat PAN and functionalized PAN-reinforced samples had an 18% and a 50% increase in fracture energy, respectively, compared to the control composite. In addition, the samples reinforced with functionalized PAN nanofibers had 27% higher interlaminar strength compared to neat PAN-reinforced composite, implying more efficient stress transformation as well as stress distribution from the matrix phase (resin-rich area) to the reinforcement phase (carbon/phase) of the composites. The enhancement of fracture toughness provides an opportunity to alleviate the prevalent issues in laminated composites for structural operations and facilitate their adoption in industries for critical applications.

## 1. Introduction

Laminated composites, i.e., carbon/epoxy composites, have been extensively utilized in a variety of structural applications owing to their superior mechanical qualities, manufacturability, and corrosion resistance when compared to traditional materials such as metals. In a variety of structural applications, such as vehicles, aerospace, ground, and wind turbines, they are considered ideal metal substitutes [1,2,3,4,5]. The performance properties of polymer matrix composites are primarily influenced by the mechanical characteristics of the composites’ individual components (matrix phase and reinforcement phase), the loading amount of each component, the matrix and reinforcement interfacial bonding, fiber alignments, and other factors [6]. The addition of reinforcements to laminated structures, in particular, increases anisotropy in the composites. The qualities of the reinforcement (fibers) and the matrix, respectively, have been found to have a significant impact on the in-plane as well as out-of-plane mechanical properties of such composites. As a result, laminated structures’ in-plane characteristics are suited for a wide range of structural applications [7]. The laminated composite out-of-plane properties are often weak due to the inferior mechanical properties of the polymer matrix relative to the reinforcement. Furthermore, due to the propagation of the created microcracks in the brittle resin-rich layer, the laminated polymer matrix composites are prone to delamination. The use of laminated composites in essential applications is limited due to this issue [6]. As a result, studies have been carried out to improve the mechanical properties of the polymer matrix with a focus on out-of-plane properties. The incorporation of carbon nanotubes [8,9], rubbery and inorganic micro/nanoparticles [10,11], and modifications to the curing process [12] are some of the tactics used. The aforementioned treatments improved the matrix mechanical properties significantly, but they had substantial obstacles that limited their use. Nanofiller distribution and dispersion, as well as an increase in resin viscosity [13], had a negative impact on the fabrication procedures, impairing the mechanical characteristics of the fabricated parts. Furthermore, changing the curing process did not result in a significant increase in the mechanical properties of the resins, according to previous studies.

Dzenis and Reneker devised a nanotechnology-based approach for strengthening composites. Since its conception, the approach has sparked a lot of scientific attention. The researchers used an electrospun nanofiber thin film between the reinforcement layers to improve resin-rich layers’ mechanical characteristics [14]. The use of electrospinning technology to fabricate nanofibers with a larger surface area than the ordinary microfibers provides a simple and cost-effective approach for improving the mechanical characteristics of the resin-rich zone with minimum influence on the fabrication procedures [6]. As a result, electrospun nanofiber interleaving has been intensively researched in recent years for enhancing the mechanical characteristics of resin-rich layers in laminated composites [15,16,17,18]. The fracture toughness of epoxy adhesives [19] and neat resins [20,21] were improved as a result of this research. Previous studies have shown that the mechanical characteristics of the laminated composites were affected by factors including the type of nanofiber and its diameter, as well as the thickness of the nanofiber mat, and the interfacial interaction between nanofibers and the polymer matrix [22]. Zhang et al. [23] showed that the G_IC_ was obtained at 547 and 749 J m^−2^ for the control composite (without nanofibers) and for the composites reinforced with poly (e-caprolactone) (PCL) nanofiber, with a 210 nm diameter, respectively. Therefore, the incorporation of PCL nanofiber led to a 37% improvement in G_IC_ Brugo and Palazzetti [24] employed a Nylon 6,6 nanofibrous mat to improve the steady-state crack propagation of a unidirectional carbon/epoxy composite. They reported that the nanofiber interleaving of the Nylon 6,6 nanofibrous mat improved the fracture toughness of the composite up to 11%.

The surface functionalization of electrospun carbon nanofibers was carried out by Chen et al. to improve the properties of the epoxy resins and the hybrid multi-scale traditional carbon/epoxy composites, including flexural properties, interlaminar shear strength, as well as impact energy absorption [25]. Glycidyl methacrylate (GMA)-modified polystyrene [26] and polyacrylonitrile [27] were researched to verify whether they have a good impact on the electrospun nanofibers and the epoxy matrix interaction.

The overarching aim of this study is to observe the effect of the addition of functionalized PAN nanofibers on the fracture toughness of a traditional carbon/epoxy composite. The GMA-grafted PAN polymer used in this study was created by a free radical reaction. The epoxy groups on the backbone of PAN nanofibers provide a better coupling site for crosslinking with other active groups in the matrix, such as amines [28,29]. However, in previous research [27], the effect of the functionalization of PAN nanofiber was investigated to observe its effect on mechanical properties, and no studies have been conducted to investigate the effect of PAN nanofiber functionalization on the fracture toughness of laminated composites. As a result, the carbon textiles were coated with functionalized PAN nanofibers. The material was then subjected to a wet-layup procedure in order to create carbon/epoxy composites. The fracture toughness of the prepared composites was then evaluated using mode I fracture energy assessment tests to see the effect of PAN chemical modification on the fracture toughness. The results were compared to those obtained for the control samples and the composite with neat PAN nanofibers [30].

## 2. Materials and Methods

### 2.1. Materials

Chemicals such as polyacrylonitrile (Mw = 150 kDa), N-dimethylformamide (DMF, 99.8%), glycidyl methacrylate (GMA, 97%), and benzoyl peroxide (BPO) were procured from Sigma-Aldrich (Saint Louis, MO, USA). Jinsor-Tech Industrial Co. (Taichung, Taiwan) provided a 300 g m^−2^ traditional unidirectional carbon fiber fabric. Hexion Inc. (Columbus, OH, USA) provided the epoxy resin (EPONTM Resin 828) as well as the curing agent (EPIKURETM Curing Agent F205).

The functionalized PAN was created using the procedures described in Neisiany et al. [27]. Using free radical polymerization, the GMA was grafted onto PAN. A round-bottomed flask was charged with 5 g of PAN and GMA at a mass ratio of 10:1. The flask was then filled with 50 mL and 0.01 g of DMF and BPO, respectively. The flask was then sealed and purged with nitrogen gas for 15 min. Subsequently, it was immersed in an oil bath at 80 °C and agitated for 15 h. The mixture of ethanol and hexane (with a ratio of 3:1) was added to the flask to precipitate the reaction mixture. To remove unreacted monomers and homopolymers, the products were finally filtered and extracted with ethanol for 12 h in a Soxhlet apparatus. The powder was then dried for 15 h in a vacuum oven at 60 ℃ and stored in a desiccator.

### 2.2. Electrospinning

**Figure 1:**
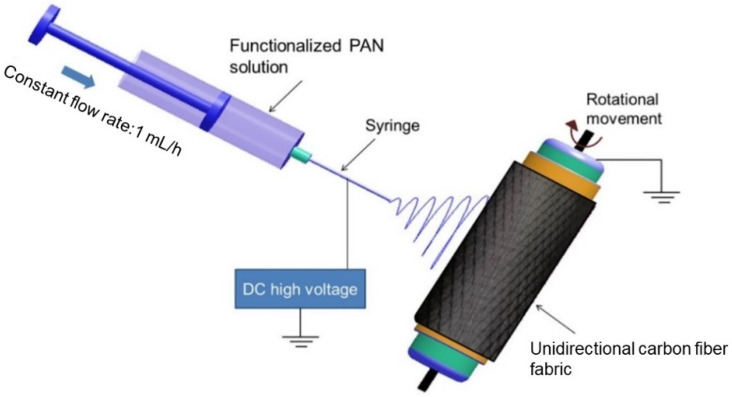
Electrospinning setup for depositing nanofibers on the surfaces of carbon fiber.

In order to prepare the electrospinning solution, 10 wt% of functionalized PAN was dissolved in DMF, while the magnetic stirring was kept for 24 h at room temperature. A 5 mL blunt syringe with a needle gauge of 21 was used to inject the solution. Before the electrospinning, the unidirectional carbon fiber fabrics were firstly wrapped around a cylindrical grounded drum with a diameter of 20 cm. Next, the electrospun functionalized PAN nanofibers were deposited directly on the surfaces of the unidirectional carbon fiber fabric. A reliable electrospinning procedure is required to produce consistent nanofibers free of beads. As a result, the process parameters were changed to match the prior work of Neisiany et al. [27]. The deposition was kept to 1 g of functionalized PAN nanofibers, which were deposited on a unit area of the unidirectional carbon textiles. The electrospinning process and the deposition of functionalized PAN nanofibers on the surfaces of carbon fiber textiles are depicted schematically in Figure 1.

### 2.3. Composite Preparation

Hand layup was used to make the carbon/epoxy composite panels, and then a Vacuum Assisted Resin Transfer Molding (VARTM) approach was used to optimize the wetting mechanism of the nanofibers and carbon fiber textiles. The epoxy resin and its curing agent were mixed with a mass ratio of 100:58.

**Figure 2:**
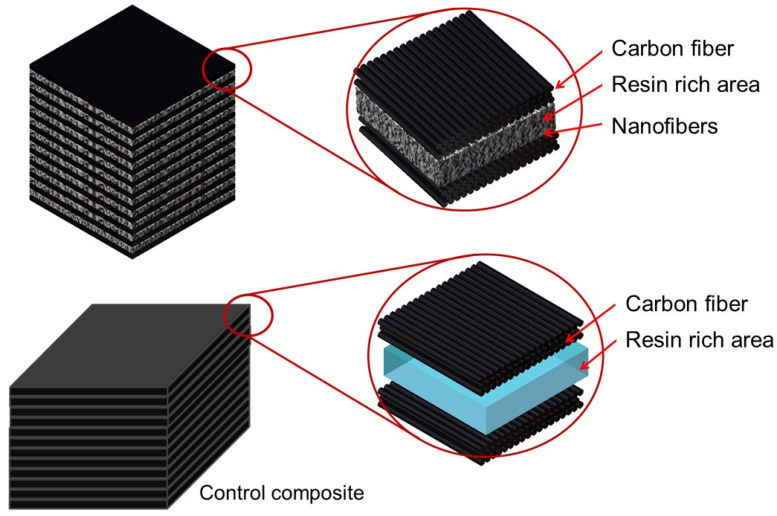
The schematic illustration of the hybrid and control composites cross-sections.

Twelve layers of uniaxial carbon fiber were used to strengthen the epoxy matrix. The reinforced epoxy matrix was held in a parallel alignment by composite panels. The nanofiber embedded panel had 11 layers of functionalized PAN nanofibers, whereas the control composite had no nanofibers at all (Figure 2). During the first curing process, a 27 mmHg vacuum pressure was applied at room temperature for 6 h and kept for 12 h to completely cure the samples. The composite panels were oven-cured for 30 min at 60 °C.

### 2.4. Characterizations

#### 2.4.1. Morphological Studies

The morphology of deposited electrospun nanofibers on the surfaces of unidirectional carbon fiber textiles, the thickness of nanofiber layers, and the surfaces of cracked composites were investigated using electron microscopy. After sputter coating the sample surfaces with a thin layer of gold, the images were captured using a Hitachi S-4300 field emission scanning electron microscope (FE-SEM) (Tokyo, Japan).

#### 2.4.2. Fracture energy studies

Water jet cut samples with lengths of 130 mm, widths of 25 mm, and crack lengths of 45 mm were obtained from composite panels for a double cantilever beam (DCB) experiment. To apply the load, steel hinges were firstly fastened to both tips of the cut test specimens. The DCB fracture tests were conducted according to ASTM D 5528 [31]. The Modified Beam Theory equation was used to determine the mode I energy release rate G_Ic_ (MBT).

$$G_{\mathrm{IC}}=3P\delta /2\mathrm{ba}$$

In this equation, P and δ are the load and the load point displacement, respectively, while b and a are the specimen’s width and the delamination length, respectively. The DCB tests were carried out by an MTS Criterion ^®^ Series 40 Electromechanical Universal Test machine (Eden Prairie, MN, USA) in triplicate under quasi-static stress using a 5 kN load cell. The displacement rate was set at 3 mm.min^−1^ while the relative humidity and temperature were kept at 60% and 20 °C, respectively. During testing, a digital camera (Canon EOS 600D with an EF 100 mm f/2.8 Macro Lens (B & H Foto & Electronics Crop., New York, NY, USA)) was utilized in order to track the crack growth at 5 s intervals.

## 3. Results and Discussion

### 3.1. Morphology of the nanofibers

**Figure 3:**
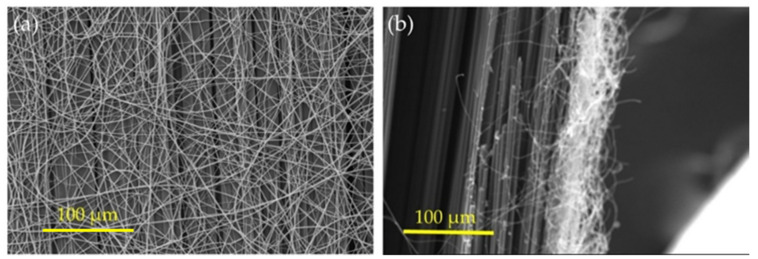
FE-SEM micrographs of (**a**) 0.05 g·m^−2^ deposition of the functionalized PAN nanofiber layer on the surfaces of unidirectional carbon fiber (**b**) the cross-section 1 g·m^−2^ deposition of functionalized PAN nanofiber layer on the surfaces of unidirectional carbon fiber.

The average diameter of the functionalized PAN nanofiber was 416 nm with a standard deviation of 93 nm, as mentioned in the previous study [27]. The morphology of the deposited functionalized PAN nanofibers on the surfaces of the unidirectional carbon fiber textiles was evaluated using the FE-SEM technique. Figure 3a shows FE-SEM micrographs after depositing 0.05 g·m^−2^ of the functionalized PAN nanofibers on the surfaces of unidirectional carbon fiber fabric. The functionalized PAN nanofibers were found to be randomly deposited on the surface of carbon fiber textiles. Furthermore, the nanofiber orientation was unaffected by collection rotation or transverse needle movement. FE-SEM micrographs were also collected to evaluate the thickness of the functionalized PAN nanofiber mat following complete deposition of them on carbon fiber fabric, which was 1 g·m^−2^. Figure 3b shows the findings of the nanofiber layer thickness assessment. The thickness of the nanofiber layers was predicted to be around 22 µm prior to the fabrication of the composite, as shown in the micrographs. The ideal thickness of the nanofiber layer, according to Chen et al., was around 20 µm. Increases in thickness above this value resulted in less resin diffusion through the nanofibers and, as a result, lower mechanical characteristics of the composite [25]. As a result, it is expected that the deposition of 1 g·m^−2^ nanofiber on the carbon fiber surfaces will provide the highest fracture toughness enhancement.

### 3.2. Fracture Energy Analysis of the Composites

**Figure 4:**
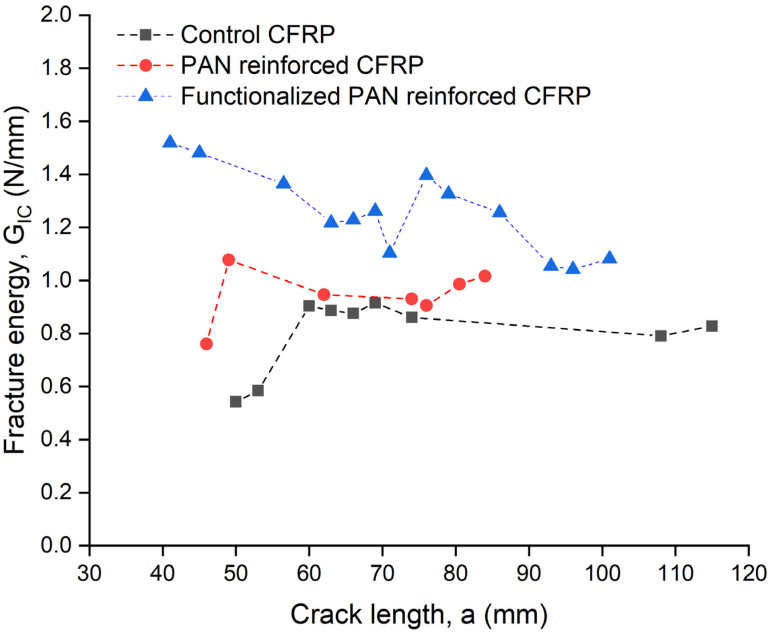
Representative R-curves of the DCB tests for control, PAN-reinforced [30], functional PAN-reinforced composites.

To determine the R-curves for various crack lengths of the samples, fracture energy was calculated. The fluctuations in fracture energy during crack propagation are depicted in Figure 4. According to the results, the fracture energies of the control materials, PAN-reinforced samples [30], and functionalized PAN-reinforced specimens were determined to be 0.832 ± 0.07, 0.981 ± 0.08, and 1.246 ± 0.12 N·mm^−^^1^, respectively. The PAN-reinforced samples had an 18% increase in fracture energy compared to the control samples, while the functionalized PAN-reinforced samples had 50% and 27% increase in comparison with the control and neat PAN-reinforced composites, respectively. Nonetheless, the fracture energy improvement of functionalized PAN-reinforced CFRP samples was greater than that of the PAN-nanofiber-reinforced CFRP samples, which could be attributable to the distinct failure processes in the samples. The improvement was reported to be 37% and 11% for PCL [23] and Nylon 6.6 [24] nanofibers, which are significantly less than 50% improvement for functionalized PAN nanofibers.

**Figure 5:**
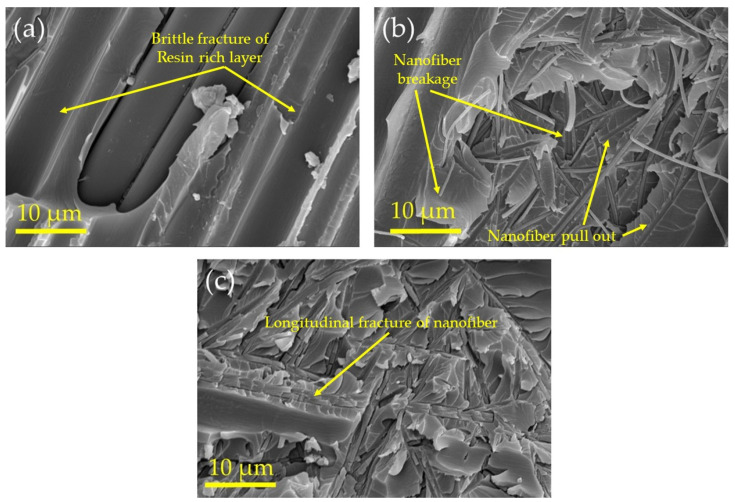
The fractured surface FE-SEM images of (**a**) the control composite, (**b**) reinforced composite interleaving with neat PAN nanofibers, and (**c**) the composite reinforced functionalized PAN nanofibers.

The failure mechanisms in various reinforcing configurations have been studied using SEM images of fracture surfaces. Figure 5 presents the FE-SEM micrographs of the shattered surface of the control sample (Figure 5a), PAN nanofiber-reinforced composites (Figure 5b), and functionalized PAN nanofibers (Figure 5c). When the fracture surface of the control sample is compared to composites reinforced with nanofiber mats, it is concluded that the control sample’s fracture surface is reasonably smooth and has oriented fracture characteristics. The microcracks propagation from the stress-concentrated locations caused this fracture [32,33]. The fracture surface roughness rose remarkably in the PAN-reinforced composites and functionalized PAN nanofiber. The presence of the electrospun nanofibers in the resin-rich layers likely prevented the sharp propagation of the microcracks, as evidenced by the roughness of fracture surfaces. This was accomplished by guiding the cracks in a more zigzag pattern, resulting in an increase in crack propagation resistance [32]. Due to nanofiber deboning and pullout from the matrix, voids, and holes appeared on the fracture surfaces of the resin-rich area in the composite reinforced with neat and functionalized PAN nanofibers. The improvement in composite fracture toughness found in previous investigations was attributed to nanofiber breaking and pullout mechanisms [33]. In addition, when comparing Figure 5b,c, it is clear that the composite fracture surfaces reinforced with functionalized PAN nanofibers are rougher than those acquired for the composite reinforced with pristine PAN nanofibers. Furthermore, the SEM images show that PAN-reinforced composites have a higher degree of nanofiber pullout, but the functionalized PAN-reinforced composite has more transverse and longitudinal nanofiber breaking. These mechanisms absorbed more energy than nanofiber pullout and demonstrated a stronger adhesion/bonding between functionalized PAN nanofibers and the epoxy matrix. As a result, functionalizing PAN nanofibers improved the interfacial interaction between the fibers and the matrix, resulting in more efficient stress distribution (within the resin-rich region) and stress transformation from the resin-rich area to the reinforcement phase (carbon fiber), which can be called the bridging effect [23]. Furthermore, the local failure around the reinforcement phase of the PAN nanofiber-reinforced samples was a combination of cohesive and adhesive failures, whereas the samples reinforced with functionalized PAN nanofibers experienced a complete cohesive failure. This discrepancy could be explained by the effect of PAN functionalization on the reinforced resin and the carbon bonding energy.

The addition of Nylon 6.6 nanofibers to unidirectional samples had an insignificant influence on the samples, according to a study by Brugo and Palazzetti [24]. The authors speculated that the break spanned plies mitigated the propagation in the same interface where a thin layer of Nylon 6.6 nanofibers was deposited. The study reported that the fracture energy of the Nylon 6.6 nanofiber-reinforced woven carbon plies was improved. For PAN and functionalized PAN-reinforced materials, similar conclusions can be drawn from the aforementioned study. Although there was a significant improvement in fracture behavior in this study, it is likely to offer much more significant gains in woven plies applications. This is something that can be investigated in future research studies.

## 4. Conclusions

The effect of integrating functionalized PAN nanofibers between carbon fiber fabrics on the laminated structural composite delamination characteristics was examined in this study. PAN nanofibers were functionalized to increase the nanofibers and epoxy resin matrix interaction, and therefore the reinforced panel’s delamination strength. Both types of reinforced panels demonstrated a considerable increase in fracture energy, but the samples reinforced with functionalized nanofibers had higher delamination strengths than the neat PAN nanofiber-reinforced samples. The presence of nanofibers delayed crack initiation by acting as a matrix reinforcement, as evidenced by the increased interlaminar fracture energy G_Ic_ The PAN-reinforced samples had an 18% increase in fracture energy compared to the control samples, while the functionalized PAN-reinforced samples had a 50% and 27% increase in comparison with control and neat PAN-reinforced composites, respectively. Furthermore, FE-SEM micrographs demonstrated that, compared to control samples, the microcracks created in functionalized PAN-reinforced composites were forced to disrupt/shatter a considerable amount of matrix during the propagation stage, needing more energy to extend. The improvement of fracture toughness for laminated structural composites will aid in the containment of cracks and prevent further fracture. This could be critical in stopping sudden and brittle failure in structures when cracks are present in the system and provide a warning so that necessary precautions can be taken.

## Data Availability

The data used to support the findings of this study are available from the corresponding authors upon request.
